# Acquired homotypic and heterotypic immunity against oculogenital *Chlamydia trachomatis *serovars following female genital tract infection in mice

**DOI:** 10.1186/1471-2334-5-105

**Published:** 2005-11-17

**Authors:** Joseph M Lyons, Servaas A Morré, Lucy P Airo-Brown, A Salvador Peña, James I Ito

**Affiliations:** 1 Department of Infectious Diseases, City of Hope National Medical Center and Beckman Research Institute, Duarte, California 91010, USA; 2 Laboratory of Immunogenetics, Section Immunogenetics of Infectious Diseases, VU University Medical Center, Amsterdam, The Netherlands; 3 Analytical Cytometry Laboratory, City of Hope National Medical Center and Beckman Research Institute, Duarte, California 91010, USA

## Abstract

**Background:**

*Chlamydia trachomatis *is the most common sexually transmitted bacterial pathogen causing female genital tract infection throughout the world. Reinfection with the same serovar, as well as multiple infections with different serovars, occurs in humans. Using a murine model of female *C. trachomatis *genital tract infection, we determined if homotypic and/or heterotypic protection against reinfection was induced following infection with human oculogenital strains of *C. trachomatis *belonging to two serovars (D and H) that have been shown to vary significantly in the course of infection in the murine model.

**Methods:**

Groups of outbred CF-1 mice were reinfected intravaginally with a strain of either serovar D or H, two months after initial infection with these strains. Cellular immune and serologic status, both quantitative and qualitative, was assessed following initial infection, and the course of infection was monitored by culturing vaginal samples collected every 2–7 days following reinfection.

**Results:**

Serovar D was both more virulent (longer duration of infection) and immunogenic (higher level of circulating and vaginal IgG and higher incidence of IgA in vaginal secretions) in the mouse genital tract. Although both serovars induced cross-reacting antibodies during the course of primary infection, prior infection with serovar H resulted in only a slight reduction in the median duration of infection against homotypic reinfection (p ~ 0.10), while prior infection with serovar D resulted in significant reduction in the median duration of infection against both homotypic (p < 0.01) and heterotypic reinfection (p < 0.01) when compared to primary infection in age and conditions matched controls.

**Conclusion:**

Serovar D infection resulted in significant homotypic and heterotypic protection against reinfection, while primary infection with serovar H resulted in only slight homotypic protection. In addition to being the first demonstration of acquired heterotypic immunity between human oculogenital serovars, the differences in the level and extent of this immunity could in part explain the stable difference in serovar prevalence among human isolates.

## Background

*Chlamydia trachomatis *is the most common bacterial pathogen associated with sexually transmitted genital tract infections both in the United States [1] and worldwide [2]. It is generally accepted that most female genital tract infections with *C. trachomatis *are both asymptomatic and without severe sequelae [3]; and, that despite improved screening programs and the availability of highly effective antibiotics [4-6], there has been a significant increase in the incidence of *C. trachomatis *genital tract infection within the last half decade [2,7]. Although epidemiologic studies suggest that prior infection with *C. trachomatis *confers some short term protection against reinfection [8,9], the exact nature of this acquired immunity remains undetermined as do issues relating to the serovar specificity and possible involvement of this immunity in the more severe sequelae associated with multiple and/or chronic infection [10-12].

It has been advanced that serovar specific immune responses, particularly those made to the major outer membrane protein (MOMP), contribute to protection, whereas responses to broadly shared antigens, particularly those induced by chlamydial heat shock protein 60 (Hsp60), are associated with the immunopathologic injury that contributes to ectopic pregnancy and tubal infertility [13-19]. There is also increasing evidence from both human epidemiologic studies and animal model experiments to support the hypothesis that a protective immune response to reinfection is a complex, site of infection variable interaction between *C. trachomatis *and specific Th1 dominant cellular responses and interferon-gamma mediated Th1 augmenting humoral responses [20,21].

In attempts to understand the salient features and specific components of this interplay, a great deal of work has been performed using slightly modified versions of a murine model of female genital tract infection first described by Tuffrey and Taylor-Robinson [22]. In our laboratory, we routinely use strains belonging to the human oculogenital biovar of *C. trachomatis *and have previously reported significant differences in the course of infection among strains belonging to 7 oculogenital serovars, which loosely correlated with the prevalence of the serovars among human clinical isolates, especially for the most and least prevalent serovars, i.e. D and E versus H and I [23].

The purpose of the present study was to expand on these observations by assessing the degree of homotypic and heterotypic protection against reinfection that follows resolution of infection with human isolates of *C. trachomatis*. Two strains were selected from the previously studied collection of strains: the strain of serovar D which was shown to establish the longest duration of infection (median duration of 38 days) and greatest humoral response; and the serovar H strain which had the shortest duration (median duration of 7 days) and lowest humoral response. In addition, lower genital tract infection with serovar D was shown to both ascend into the uterine horns with a greater frequency than serovar H, as well as shed more infectious units during the acute phase of infection [23]. In a separate study we demonstrated a link between certain in vitro growth characteristics and differences in the level of cytotoxic activity associated with elementary bodies between these strains [24]. Thus, strain selection was made with the intention of reflecting the greatest diversity observed among the strains studied in the murine model, as well as to represent serovars that have significantly different prevalence rates among human isolates in the hope that the results of the study would provide some insight into the possible causes of these differences. Duration of genital tract infection was used to determine the extent of protection, and humoral and cellular immune response data were evaluated to identify factors that associated with any observed protection.

## Methods

### Animals

Outbred CF-1 female mice (Charles River Labs) were purchased at 7 weeks of age and were allowed to acclimate for one week prior to use. All experiments were conducted in a BL-2 containment facility in compliance with animal care regulations and under protocols approved by the institutional research animal care committee.

### Bacteria and culture technique

Mycoplasma-free and pure PCR-typed strains of serovar D and H [23,24] were propagated, purified, titrated, and isolated in cycloheximide treated McCoy cell monolayers using standard techniques. Separate vials of the same -70°C stored stocks were used for both infections.

### Murine model

Progesterone, in the form of medroxyprogesterone acetate (Depo-Provera, Upjohn), was given subcutaneously (sc) in 2.5 mg doses, 10 and 3 days prior to infection [22,23]. Prior to infection, vaginal specimens were obtained for culture and cytology, and 2 hours later mice were inoculated intravaginally by direct instillation of 25 μl of sucrose phosphate buffered transport medium (SP) containing 1–3 × 10^7 ^inclusion forming units (ifu).

### Sample collection

The presence of *Chlamydia *in the lower genital tract was determined by swabbing the vaginal vault and ectocervix with a calcium alginate swab, which was cultured for the organism. Plasma and vaginal secretions were obtained prior to infection. Blood was taken from a small tail vein incision, diluted 1:10 in PBS, and the plasma (P) was separated by centrifugation. Vaginal secretions (VS) were collected over a two-hour period by absorption into a piece of surgical sponge, and eluted into 400 μl of PBS. All samples were frozen at -20°C until tested.

### Serological analyses

Anti-chlamydial IgG and IgA titers in blood and vaginal secretions were determined by indirect solid phase enzyme immunoassay (EIA) using SDS solubilized Ct elementary bodies as antigen. Western immunoblot analysis were performed on a similar antigen preparation which had been SDS-PAGE separated and transferred to nitrocellulose paper. After incubation with sample (P at a final dilution1:200; VS at a final dilution of 1:20), reactive bands were visualized with standard EIA reagents.

### Spleen cell analysis

Cellular immune responses were determined using a standard assay for ^3^H-thymidine (^3^H-Td) incorporation. Specific responses were measured using formalin preserved elementary bodies (EB) at a ratio of 100 EB:1 spleen cell, and non-specific responses measured using concavalin A (Con A) at 2 μg/ml, phytohemagglutinin (PHA) at 5 μg/ml, and lipopolysaccharide (LPS) at 100 μg/ml. Single-cell suspensions were prepared by gently pressing the spleen through a nylon sieve. Debris was allowed to settle for 2 min, and the supernatant containing single cells was spun down at 500 × g for 10 minutes. Erythrocytes were lysed with NH_4_Cl solution and cells were washed three times with RPMI 1640 medium containing 10% fetal bovine serum and plated in triplicate, in 96-well plates at a concentration of 5 × 10^5 ^cells per well. Proliferative responses were measured by uptake of 1 μCi of ^3^H-thymidine (^3^HTd) per well for the last 24 hours of a 72 hour incubation period. As a measure of response, a stimulation index (SI) was calculated (SI = ^3^H-Td incorporation with stimulation/^3^HTd incorporation without stimulation).

### Statistical evaluation

Duration of infection data were analyzed by the Wilcoxon Rank Sum Test; meaned data by F-Test; and frequency data by Chi Square.

## Results

### Homotypic and heterotypic protection against reinfection

Consistent with our previous report [23], intravaginal inoculation with the serovar D strain resulted in a longer duration of infection in previously uninfected mice compared with the serovar H strain (22.5 days versus 15.5 days, p = 0.05) (Table 1). Analysis of the effect of prior *C. trachomatis *genital tract infection on the duration of infection following homotypic and heterotypic reinfection showed that infection with serovar H resulted in only slight homotypic protection (15.5 days versus 10 days duration of infection: p = 0.1), while serovar D infection resulted in significant homotypic (22.5 days versus 12 days duration of infection: p < 0.01) and heterotypic protection (15.5 days versus 5 days duration of infection: p < 0.01) against reinfection (Table 1).

**Table 1:** Acquired Homotypic and Heterotypic Immunity Against Oculogenital *Chlamydia trachomatis *Serovars Following Female Genital Tract Infection in Mice

Infection Serovar		Number of Animals	Number of Animals Culture Positive on Reinfection Day																		Median Duration of Infection	Wilcoxon Rank Sum p Value
Initial^1^	Reinfection		2	4	6	8	10	14	17	21	24	28	31	35	38	42	45	48	52	55		
D	H	12	12	5	3	2	5	2	1	0	0	0	0	0	0	0	0	0	0	0	5	<0.01
H	H	12	12	11	8	8	10	3	1	2	0	0	0	0	0	0	0	0	0	0	10	~0.10
None	H	12	12	12	12	12	11	6	5	2	2	1	0	0	1	0	0	0	0	0	15.5	--
																						
D	D	12	12	9	7	9	7	5	4	2	2	1	0	0	0	0	0	0	0	0	12	<0.01
H	D	12	12	12	12	12	11	6	10	7	7	4	1	2	1	0	0	0	0	0	24	NS
None	D	12	12	12	12	12	11	7	5	6	4	4	2	1	1	2	0	0	0	0	22.5	--

^1^Median duration of infections during the primary infection phase of this study were 10 days with serovar H and 33 days with serovar D.

### Humoral immune responses

#### Quantitative

As in our previous study [23], genital tact infection with serovar D resulted in a significantly greater quantitative anti-chlamydial humoral response compared to the response to serovar H infection (Table 2). Serovar D induced significantly higher plasma (p < 0.05) and vaginal (p < 0.05) IgG levels as compared to serovar H. Although no quantitative differences between the IgA responses following infection with either serovar D and H were observed in the vaginal secretions of animals with detectable levels of antibody, a significant difference was observed in the frequency of IgA positive vaginal secretions from serovar H and D infected animals prior to reinfection (11/24 for H vs 21/24 for D, p < 0.01, data not shown). In all cases, similar homologous and heterologous antibody titers were detected against both antigen preparations used in the assay.

**Table 2:** Serologic Analysis of Plasma and Vaginal Secretions Following Infection and Immediately Prior to Reinfection with *Chlamydia trachomatis *Serovars D and H

			Plasma and Vaginal Secretion Titers (Log _2_) (p Value)					
Infection Serovar		Number of Animals	Plasma IgG		VS IgG		VS IgA^1^	
Primary	Secondary		H	D	H	D	H	D
D	H	12	13.0	13.6	8.3	8.7	7.2	8.3
			(<0.05)		(<0.05)		(NS)	
H	H	12	11.0	11.2	5.3	6.1	6.7	6.8
None	H	12	<6	<6	<2	<2	<2	<2
								
D	D	12	12.5	13.1	7.8	8.3	7.3	7.6
			(<0.05)		(<0.05)		(NS)	
H	D	12	11.4	11.4	5.3	5.5	6.0	6.4
None	D	12	<6	<6	<2	<2	<2	<2

^1^Values listed are the mean titers of IgA positive animals only.

#### Qualitative

Tables 3 and 4 contain the Western immunoblot analysis of plasma and vaginal secretions from representative animals following resolution of initial infection and immediately prior to reinfection with serovar H (Table 3) or with serovar D (Table 4). Consistent with the quantitative findings, plasma and vaginal secretions from serovar D infected mice contained antibodies to a greater array of antigens than specimens from serovar H infected mice; and although often more intense when homologous, plasma from mice infected with either serovar gave similar immunoblot patterns against both antigen preparations, thus demonstrating the induction of a high level of cross-reacting IgG during infection.

**Table 3:** Serological Analysis of Plasma and Vaginal Secretions from Representative Animals Following Primary Infection with Either *Chlamydia trachomatis *Serovar H or D and Immediately Prior to Infection with Serovar H

	H Primary Infection																								D Primary Infection																							
Animal	J1				H3				J3				L1				H4				J2				R1				X1				X2				R4				T2				Y4			

	Infection Duration (Days)																																															

Primary	24				24				8				24				6				24				38				42				24				42				28				42			
Secondary	2				4				10				10				21				21				2				2				4				6				14				17			

	Plasma and Vaginal Secretion Titers (Log 2) and IgG Immunoblot Reactions Against serovar H and D																																															

	H		D		H		D		H		D		H		D		H		D		H		D		H		D		H		D		H		D		H		D		H		D		H		D	
P IgG	14		13		13		13		7		8		13		13		7		8		13		13		12		13		13		14		13		13		14		14		13		13		12		13	
V IgG	8		8		8		8		2		3		9		9		<2		<2		8		9		7		8		8		7		8		9		8		8		8		8		8		8	
V IgA	9		8		8		8		<2		<2		8		8		<2		<2		4		4		5		7		8		9		7		8		7		8		8		9		8		9	

MW (kD)	P	V	P	V	P	V	P	V	P	V	P	V	P	V	P	V	P	V	P	V	P	V	P	V	P	V	P	V	P	V	P	V	P	V	P	V	P	V	P	V	P	V	P	V	P	V	P	V

190									-	-	-	-				-		-		-																												
140																																																
120																													·	-			·	-														
115																																																
110																																	·	·														
108																																																
106																																	•	•	•	•												
102																																																
100																																			-	·												
96																																																
92																					·	-															•	·										
90																																																
78																													·	-																		
72																																																
66																	·	-															·	-											•	·		
64																																																
62	·	-	·	·	•	·	·	•													•	-	•	·	·	·	•	·	•	·	•	·	·	-	·	·					•	·	•	·				
61.5																																																
60	●	·	•	•	•	·	•	•													·	-	·	-	•	•	●	•	•	•	•	•	•	•	•	•	•	●	•	•	●	•	•	•	●	•	•	•
58																													·	-																		
56																													·	-																		
50																	·	-	·	-																									·	-		
46.5	•	·			●	•	·	-					•	·	·	-					●	·			·	-	●	•	•	•	•	•	•	·	•	•			•	•			•	·	·	-	●	●
40																																																
38																																																
36																					·	-	·	-					·	-																		
33																																																
29															·	-											·	-	·	-							•	•	•	•					·	·	•	•
26																																																
22.5																										•	-																					
19.5																																																
15.5	·	·	·	·																																												

- = No reaction

Plasma = P

· = Barely visible

Vaginal secretions = V

• = Weak

● = Strong

**Table 4:** Serological Analysis of Plasma and Vaginal Secretions from Representative Animals Following Primary Infection with Either *Chlamydia trachomatis *Serovar H or D and Immediately Prior to Infection with Serovar D

	H Primary Infection																								D Primary Infection																							
Animal	I1				G3				K2				G2				K3				I4				Z1				S4				Q4				Z2				Q2				S1			

	Infection Duration (Days)																																															

Primary	6				10				6				31				14				6				45				21				14				45				52				10			
Secondary	10				17				24				28				35				38				2				4				10				14				24				28			

	Plasma and Vaginal Secretion Titers (Log _2_) and IgG Immunoblot Reactions Against serovar H and D																																															

	H		D		H		D		H		D		H		D		H		D		H		D		H		D		H		D		H		D		H		D		H		D		H		D	
P IgG	11		11		13		13		11		11		12		12		9		9		13		11		14		14		12		13		11		11		13		14		14		14		11		11	
V IgG	5		5		7		6		2		4		6		7		4		4		5		5		11		11		7		7		7		8		9		10		9		9		4		5	
V IgA	<2		<2		<2		<2		<2		<2		7		8		<2		<2		<2		2		3		5		<2		<2		4		6		8		9		10		9		<2		<2	

MW (kD)	P	V	P	V	P	V	P	V	P	V	P	V	P	V	P	V	P	V	P	V	P	V	P	V	P	V	P	V	P	V	P	V	P	V	P	V	P	V	P	V	P	V	P	V	P	V	P	V

190		-		-								-					-	-	-	-																												
140																																																
120																													·	-																		
115																																																
110																																																
108																																																
106																																					·	-			·	-						
102																																																
100																																																
96																																																
92																																	-	·														
90																																																
78																																									·	-						
72																																																
66													·	-																																		
64																																																
62					●	·	•	-					•	·	•	•									·	·	•	·	·	·	·	·			·	-	●	·	●	·	●	●	●	●				
61.5																																																
60					●	•	●	•	·	-	·	-	•	·	•	·									●	•	•	•	•	·	•	•	·	-	·	·	•	·	•	-	•	•	•	•	·	·	•	·
58																																																
56																																																
50																																																
46.5	·	-	·	-	●	•			•	·			•	·	•	·					•	·	·	-	●	·	•	·	·	-	●	•	·	-	•	•	•	·	•	·	·	-	●	•	·	-	•	·
40																																																
38																																	-	·														
36																																					•	·	•	•	·	-	·	-				
33																																																
29					·	-	·	-																											-	·	•	·	•	•	·	-	·	-	·	·	•	·
26																																																
22.5																													·	-									·	-								
19.5																																			·	-	·	·					·	-				
15.5	·	·	·	·																																												

- = No reaction

Plasma = P

· = Barely visible

Vaginal secretions = V

• = Weak

● = Strong

With the exception of the link between quantitative humoral response and protection, statistical analysis of the serologic and duration of infection data did not detect a humoral factor(s) that correlated with the shorter duration of infection observed following reinfection.

### Splenic lymphocyte responses

Table 5 summarizes the chlamydia-specific and mitogen non-specific splenic lymphocyte responses obtained from groups of four animals 55 days following genital tract inoculation with either serovar H or D. Mean responses to PHA and both elementary body preparations were greater following infection with either serovar when compared to non-infected controls (p 0.05); while responses to both Con-A and LPS were not different from controls. No association could be found between a particular pattern or magnitude of cellular responses and an individual animals duration of infection.

**Table 5:** Splenic Lymphocyte Responses 55 Days After Primary Infection with Serovars H and D

	Mean Stimulation Index ± 1 SEM				
	EB Serovar		Standard Mitogens		
Group	H	D	Con-A	PHA	LPS
Control	13.0 ± 1.7	15.9 ± 1.8	79.5 ± 12.0	6.2 ± 1.8	58.4 ± 5.3
H	19.2 ± 3.1*	23.4 ± 4.1*	96.2 ± 15.6	11.3 ± 6.4*	58.7 ± 15.8
D	19 ± 1.5*	21.7 ± 1.7*	78.8 ± 6.8	11.9 ± 6.4*	51.1 ± 6.3

*Significantly different from Control mean at p 0.05

## Discussion

Using a murine model of *C. trachomatis *female genital tract infection, we have demonstrated that homotypic immunity against reinfection was induced following initial infection with either serovar D or H, but that a more significant level of protection was observed following infection with serovar D. However, heterotypic protection against reinfection was strain dependant and was seen only when the initial infection was with the more virulent and immunogenic strain of serovar D. These results are the first demonstration of heterotypic immunity between two oculogenital serovars following female genital tract infection in the mouse model, as well as being the first comparative study that suggests a possible strain dependent restriction on the process. The findings are consistent with other studies that have observed heterotypic immunity in the context of MoPn and serovar E. Also consistent with prior reports is the correlation between the level of protection observed and the virulence of the strains used to infect the mouse genital tract, with the more virulent MoPn inducing a more solid level of heterotypic immunity against serovar E than was observed in the reverse situation [25,26]. Finally, the observed differences in heterotypic and homotypic responses between serovars D and H could explain in part the relatively stable differences in the frequency of these two serovars among human isolates from different geographic locations [27-33], i.e. a prior infection with a more virulent/immunogenic serovar would confer a greater degree of protection against infection with a less virulent/immunogenic serovar, thus reducing the incidence of the latter by reducing the efficiency of serial transmission.

Consistent with human epidemiologic data [8,9], the protection observed in this model against reinfection with oculogenital serovars of *C. trachomatis *was not complete, but rather acted in a way that reduced the duration of infection and level of bacterial shedding (data not shown) during infection. With the notable exception that serovar H infection resulted in a lesser quantitative and qualitative humoral response, a thorough analysis of the individual and collective data was unable to identify any specific element(s) that correlated with protection. This finding is consistent with a report in which a similarly extensive analysis of cellular and humoral responses was performed in a comparison of the acquired immunity induced by infection with MoPn and serovar E [25]. Although not proof, this does support the current working hypothesis that acquired immunity to *C. trachomatis *female genital tract infection is a complex and integrated phenomena that relies on both Th1 and Th2 type responses made during the course of infection, which in turn enhance innate immune responses upon reinfection [20]. This complexity, which likely arises out of the ever changing physiologic and immunologic milieu within and between anatomically distinct but connected regions of the female genital tract, may account for the difficulty identifying specific components of what may be a flexible pattern of responses that lead to a given individual's level of protection against or risk of severe upper genital tract pathology [21].

Women with recurrent *C. trachomatis *infection are at increased risk of reproductive sequelae, including pelvic inflammatory disease, ectopic pregnancy and tubal infertility [10-12], which have been linked to both cellular and humoral immune responses induced during infection [13-19]. How the nature and level of homotypic and/or extent of heterotypic immunity in the murine model extrapolate to the risk of upper genital tract pathology and infertility was not assessed in this study, and is an area of investigation that has yet to be systematically addressed. Most of the experimental data relating to the severe sequelae associated with *C. trachomatis *female genital tract infection has been obtained in studies using *C. muridarum*, MoPn [34,35]. As a result, it has not been possible to clearly assess the immunologic features that are thought to contribute to severe sequelae within the human female genital tract, because the damage that occurs within the murine genital tract following infection with MoPn is a consequence of acute and not chronic infectious processes and/or recurrent infection [36,37]. Typically in the mouse and in most women, human oculogenital serovars are limited in their ability to ascend with any major pathologic consequence from the initial site of infection within the lower genital tract. However, infection of female C3H/HeN with a strain of serovar E has been shown to ascend and cause infertility without gross pathology at a low incidence following a single infection and with increased incidence upon reinfection [26]. Of particular interest is that none of these mice developed hydrosalpinx, which is the hallmark of upper tract infection with MoPn, indicating a possible alternative mechanism for the induction of infertility, one different from the tubal dilation, scarring and associated hydosalpinx that occurs as a sequelae to MoPn infection. Although no specific immunologic mechanism was identified as contributing to the development of infertility, it was noted that the C3H/HeN mouse is an Hsp60 responding strain and that Hsp60 responding strains of mice are more susceptible to MoPn induced infertility while non-responders can be infected but do not experience severe upper tract pathology. It will be worthwhile to determine if the two *C. trachomatis *strains used in the present study show a similar relationship to each other in C3H/HeN mice and whether homotypic and/or heterotypic elements of immunity play a role in the progression of events that lead to severe upper tract pathology, which is essentially the reason why *C. trachomatis *genital tract infections are significant and why intervention and prevention strategies are needed.

## Conclusion

In conclusion, we demonstrate in the first study comparing phenotypically different strains representing two human oculogenital serovars that both the level of homotypic protection against reinfection and the ability to confer heterotypic protection correlated with the virulence/immunogenicity of the strain. Extrapolating the results to human epidemiologic data could explain in part the relatively stable differences in the frequency between the most and least prevalent serovars based on a serovars ability to induce or not induce heterotypic immunity. Although no specific cellular or humoral factor(s) could be identified that associated with the observed protection, it is clear that heterotypic immunity can be induced and that the systematic study of human oculogenital serovars in the mouse model of female genital tract infection could provide information that leads to an understanding of what distinguishes a protective from an immunopathic response to infection.

## Competing interests

The author(s) declare that they have no competing interests.

## Authors' contributions

Joseph M. Lyons participated in the design, planning, execution and supervision of the study, and drafted original and final manuscripts. Servaas A. Morré analyzed the data and constructed the tables that appear in the manuscript. Lucy P. Airo-Brown performed the animal experiments and developed the serologic assays used in the study. A. Salvador Peña provided direction during the development of the integrated approach to the study of the *Chlamydia trachomatis *infections of the female genital tract of which this report is a part. James I. Ito planned and approved the study design, and participated in the preparation of the manuscript. All authors read and approved the manuscript.

## Pre-publication history

The pre-publication history for this paper can be accessed here:


